# Effectiveness of Mobile Health Interventions for Reducing Sitting Time in Older Adults: Systematic Review and Meta-Analysis

**DOI:** 10.2196/60889

**Published:** 2025-05-08

**Authors:** Siqing Chen, Chenchen Wang, Albert Ko, Carol Ewing Garber, Edward Giovannucci, Yuting Yang, Matthew Stults-Kolehmainen, Lili Yang

**Affiliations:** 1 Department of Nursing School of Medicine, Sir Run Run Shaw Hospital Zhejiang University Hangzhou, Zhejiang Province China; 2 Department of Nursing The Fourth Affiliated Hospital of School of Medicine, International School of Medicine, International Institutes of Medicine Zhejiang University Yiwu China; 3 The Faculty of Nursing Zhejiang University School of Medicine Hangzhou China; 4 Department of Epidemiology Harvard T.H. Chan School of Public Health Boston, MA United States; 5 Department of Biobehavioral Sciences Teachers College Columbia University New York, NY United States; 6 Division of Digestive Health Yale New Haven Hospital New Haven, CT United States

**Keywords:** older adults, sedentary behavior, health behavior, community, systematic review, meta-analysis

## Abstract

**Background:**

Mobile health (mHealth) provides health information through electronic devices, even at home. The escalating prevalence of sedentary behaviors among older adults, which leads to increased adverse health outcomes, underscores the pressing need for a comprehensive understanding of the effectiveness of mHealth interventions.

**Objective:**

This study aims to examine the effectiveness of mHealth interventions in the sitting time of older adults (age 55 years).

**Methods:**

A systematic review and meta-analysis of randomized controlled trials was conducted to evaluate the effects of mHealth interventions on total sitting time during waking hours, excluding sleep. A literature search was conducted using multiple databases, including PubMed, Embase, Web of Science, and Cochrane, covering articles published from the inception of each database through October 2023. The PRISMA (Preferred Reporting Items for Systematic Reviews and Meta-Analyses) guidelines were explicitly applied to structure this report. Methodological quality was assessed using the Cochrane Risk of Bias (ROB 2) tool for randomized controlled trials and the Methodological Index for Non-Randomized Studies (MINORS) tool for nonrandomized studies. Two independent reviewers screened the studies, extracted data, and assessed methodological quality using established criteria. Meta-analyses were performed using Review Manager (version 5.4; Cochrane Collaboration).

**Results:**

Ten studies were identified, of which 3 were included in the meta-analysis, while the remaining 7 were assessed exclusively in the systematic review. The interventions predominantly took place in community settings (n=3) and home-based settings (n=3). Three studies aimed to decrease sedentary behavior and 7 aimed to increase physical activity. The interventions were primarily conducted once daily (n=7) via mobile devices such as smartphones (n=7) and typically involved a single intervention delivered at different time intervals, such as every 15, 20, or 30 minutes (n=4). The interventions typically lasted 12 weeks (n=4) and used objective assessment tools, such as the ActiGraph GT3X+ (n=8). The included studies applied the habit formation theory (n=1), the self-efficacy theory (n=1), the social cognitive theory (n=1), and the social-ecological theory (n=1) as frameworks. Additionally, behavior change techniques, including “goal setting,” “problem-solving,” “action planning,” and “review behavior goal(s)” (n=6), were used. Meta-analysis of the 3 studies included showed a significant decrease in sedentary behavior with mHealth interventions compared with conventional or no interventions (weighted mean difference [WMD]=59.1 min/d, 95% CI 99.1 to 20.2; *P*=.003).

**Conclusions:**

mHealth interventions effectively reduce sitting time in older adults. Strategies using interventions with specific frequencies and durations, dedicated mobile monitoring devices, and behavior change techniques showed the potential to reduce sedentary behavior among older adults. These results also underscore the potential of mHealth as a key tool for promoting the well-being of older adults through technology-driven public health efforts.

**Trial Registration:**

PROSPERO CRD42023443926; https://www.crd.york.ac.uk/PROSPERO/view/CRD42023443926

## Introduction

In recent years, the global population of older adults has been rapidly increasing, with projections estimating that it will double to approximately 2 billion by 2050 [1]. This demographic shift is particularly pronounced in China [2]. Individuals aged 60 years and more account for 18.9% of the global population [2]. Within this group, 75% of individuals have at least one chronic health condition and 43% experience multiple comorbidities [1].

Sedentary behavior is characterized by activities with low energy expenditure (≤1.5 metabolic equivalents), while in a sitting, reclining, or lying posture [3]. Prolonged sedentary behavior is associated with an increased risk of chronic diseases, such as cardiovascular disease, type 2 diabetes, and chronic liver disease, as well as a higher all-cause mortality rate [4-6]. Furthermore, it contributes to cognitive decline, muscle loss, and premature death [7-9]. However, current strategies to promote healthy aging often prioritize physical activity while overlooking a sedentary lifestyle [8,10,11], which is an independent risk factor for adverse health outcomes [12].

Older adults spend more time engaging in sedentary behavior and less time in physical activity compared to younger adults [13-15]. The time they spend on sedentary entertainment activities is increasing, with estimates suggesting it accounts for up to 80% of their waking hours [13]. Studies show that nearly 70% of older adults remain sedentary for up to 8.5 hours per day [16,17]. Against this backdrop, reducing sedentary behavior in older adults has been listed as a priority on the public health agenda by the World Health Organization (WHO), which urges countries to develop targeted interventions [13].

Traditional interventions for sedentary behavior have focused on reducing overall sedentary time and increasing the frequency of interruptions [18]. These interventions often involve advice from health care professionals and health educational materials. However, these methods have limitations due to both individual barriers (such as mobility difficulties for older adults, lack of time, and motivation) and external barriers (such as limited access to professionals, limited intervention coverage, and high monitoring costs associated with health care professionals) [19]. Consequently, many face-to-face traditional interventions face implementation difficulties and fail to achieve reductions in sedentary behavior [20].

The WHO defines mobile health (mHealth) as the provision of health care and health information to health care professionals and the public through various mobile electronic devices [21]. With the rapid development of information technology and the widespread adoption of smart devices, mHealth interventions based on behavioral principles have emerged and become prominent tools for rehabilitation and health behavior promotion [18]. Although mHealth interventions generally tend to underrepresent older adults, the increasing functionality and user-friendliness have led to their growing popularity in this demographic. In developed countries, 61% of people aged 65 years and above own a smartphone, and the number of users continues to increase [22].

The behavior change technique (BCT) taxonomy by Michie et al [23] systematically comprises 93 hierarchically clustered techniques that can be used to develop mHealth apps (MHAs) and implement various interventions to reduce sedentary behavior [23]. Compared with traditional intervention methods, mHealth interventions combine MHAs, wearable devices, the internet, and BCTs (such as goal setting, planning, feedback, rewards, social support, and social comparison), enabling continuous, real-time assessment and monitoring of sedentary behavior, medication use, social interaction, and physiological indicators throughout the day [24].

The specific impact and underlying mechanisms of mHealth interventions on older adults’ sedentary behavior are poorly understood. To address these uncertainties, we aimed to (1) conduct a comprehensive systematic review to compare study characteristics, including country, participant demographics, disease types, sample sizes, intervention settings, duration and frequency, monitoring devices, sedentary behavior assessment tools, behavior change theories, and BCTs; and (2) explore the effects of mHealth interventions on reducing sedentary behavior in this demographic through a meta-analysis of mHealth interventions.

## Methods

### Study Design

This systematic review and meta-analysis followed PRISMA (Preferred Reporting Items for Systematic Reviews and Meta-Analysis) guidelines, as shown in Multimedia Appendix 1 [25], and was registered with PROSPERO (Prospective Register of Systematic Reviews; CRD42023443926).

### Search Strategy

The search strategy was developed based on a thorough review of existing literature and the collective expertise of our research team. The team members had undertaken specialized coursework in systematic reviews and meta-analyses and participated in multiple meta-analysis projects. Under the guidance of the principal investigators (LY, CEG, and EG), we performed exhaustive search in PubMed, Embase, Cochrane Library, and Web of Science, covering publications from the start of each database until October 2023.

Our search methodology combined specific keywords and Medical Subject Headings (MeSH), such as “sedentary behavior,” “mHealth interventions,” and “older adults,” as detailed in Multimedia Appendix 2.

### Inclusion and Exclusion Criteria

Eligible studies met the criteria listed in Textbox 1.

**Eligibility criteria.**

**Inclusion criteria**

Population: older adults (age≥55 years), consistent with definitions used in previous studies [18,26,27]

Intervention: mobile health intervention, defined as using any form of electronic devices such as smartphones, smartwatches, iPads, the internet, and related digital technology to promote health service

Comparison: not exposed to any mHealth interventions or conventional care (qualitative studies and pre-post designs are exempt from this criterion)

Primary outcomes: total sitting time during waking hours, excluding sleep duration (such as minutes spent sitting per day)

Study design: randomized controlled trials, pilot studies, study protocols, and pre-post studies

**Exclusion criteria**

Not published in English

Incomplete studies (such as ongoing studies or lacking necessary data for calculating an effect size for the meta-analysis)

### Study Selection and Data Extraction

All studies identified were uploaded to the Endnote X9 library (Clarivate) and underwent deduplication. Next, independent reviewers (SC and YY) independently screened the identified studies’ titles, abstracts, and full texts for potential eligibility based on predefined inclusion and exclusion criteria. After discussion and consensus, irrelevant articles were excluded. Data were then extracted using a standardized Microsoft Excel (version 16.78.3) spreadsheet. We extracted essential details such as author, year, country, participant ages, participant gender, disease, study type, sample size, intervention setting, intervention duration, intervention device, sedentary behavior assessment tools, primary assessment metrics, theories, and BCTs used in the study. Two reviewers performed data extraction independently and cross-checked each selected study to validate accuracy. Discrepancies in the screening process were resolved through consensus or consultation with a third reviewer (LY).

### Risk of Bias Assessment

The methodological quality of the included studies was assessed using the Cochrane Risk of Bias (ROB 2) tool for RCTs and the Methodological Index for Non-Randomized Studies (MINORS) tool for nonrandomized studies. For RCTs, 5 domains were evaluated using ROB 2 by the Cochrane Handbook [28]: randomization process, deviations from intended interventions, missing outcome data, measurement of the outcome, and selection of the reported result. Each domain was rated as low risk, some concerns, or high risk of bias. For nonrandomized studies, MINORS was used to assess 12 items (eg, clearly stated aim, prospective data collection, and unbiased end-point assessment), each given a score of 0 (not reported), 1 (reported but inadequate), or 2 (reported and adequate) [29]. For noncomparative studies, 8 items are assessed, with a maximum score of 16. Scores between 13 and 16 indicate high quality, 9-12 moderate quality, and 5-8 low quality [29,30]. Two researchers (SC and AK) independently conducted the assessments, with cross-checking results. Disagreements were resolved through discussion or consultation with a third reviewer (LY).

### Statistical Analysis

Summary estimates were calculated for primary outcome variables measuring sedentary behavior, such as minutes spent sitting per day. The inverse variance method was applied using both random-effects and fixed-effects models based on the level of heterogeneity among studies [31]. When outcome measures across studies varied in terms of units or methods, standardized mean differences with 95% CIs were calculated based on mean values and SDs. Conversely, when studies reported outcomes using consistent measurement units and methods, mean differences were used to compare the intervention effect directly. When mean values and SDs were not reported, the missing data were obtained by contacting the original authors.

Meta-analyses were performed using Review Manager v5.4 (Cochrane Collaboration) [28], and the *I*^2^ statistic was used to assess heterogeneity across the studies. A fixed-effect model was used when heterogeneity was not significant (*I*^2^50%), while a random-effects model was used if significant heterogeneity was present (*I*^2^>50%).

## Results

### Search Selection

**Figure 1:**
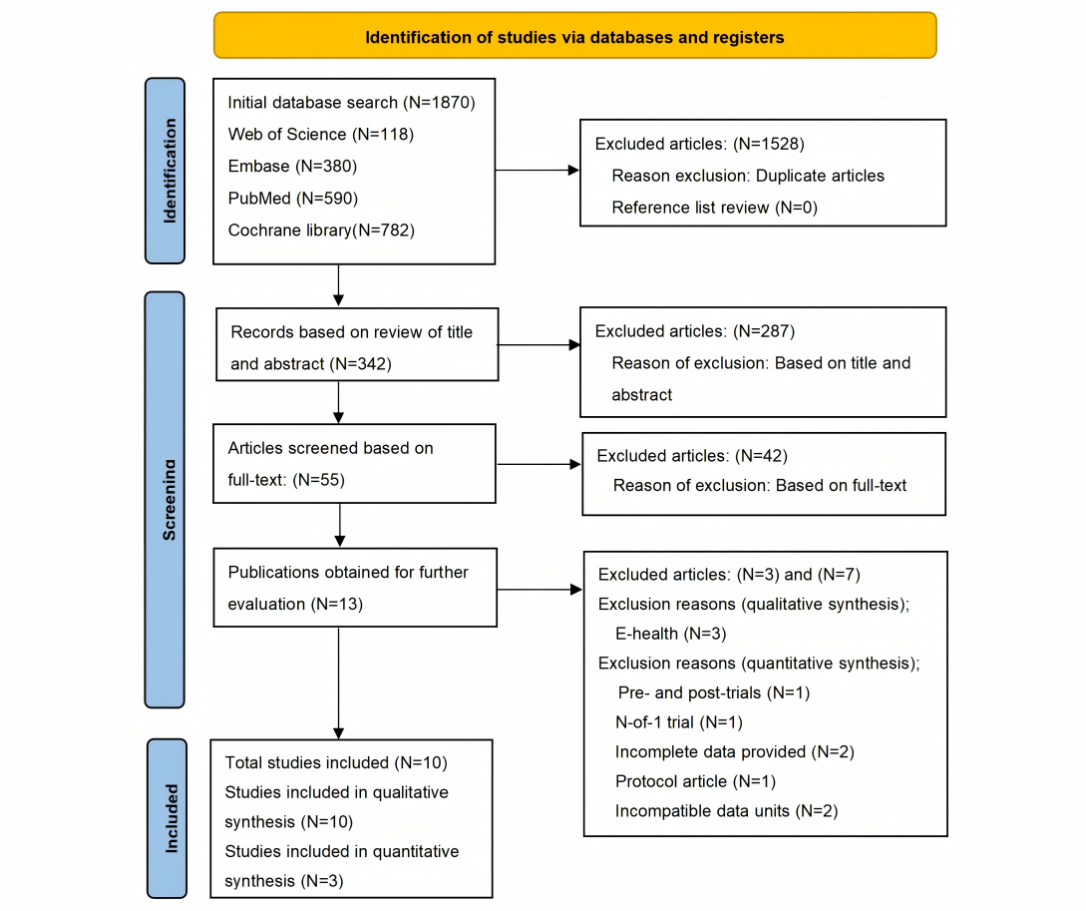
PRISMA flow diagram of study selection and identification.

The PRISMA flowchart in Figure 1 details the process of identifying eligible studies. The initial database search identified 1870 articles from Web of Science (n=118), Embase (n=380), PubMed (n=590), and Cochrane (n=782). After removing duplicates, 1528 articles were excluded. Further screening of titles and abstracts removed 287 articles that did not meet the inclusion criteria. A full-text review led to an additional 42 articles being excluded due to their failure to meet the necessary criteria, leaving 13 articles for analysis. Three articles explicitly related to eHealth were excluded [32-34], resulting in 10 articles for systematic review [35-44]; 7 of 10 articles were excluded from the meta-analysis. The reasons for exclusion were incomplete data in 2 articles [36,38], pretrial and posttrial design in 1 article [42], 1 article being a study protocol [39], 1 N-of-1 trial [43], and incompatible data units in 2 studies [35,40]. Ultimately, 3 articles [37,41,44] were included in the meta-analysis.

### Study Characteristics

The studies included in this review were published between 2015 and 2022 [35-44], and they were conducted in various countries, predominantly in the United States (n=6), Canada (n=2), and Spain (n=2). Participants’ ages ranged from 55 to 89 years, with 4 studies focusing on individuals aged ≥60 years, and 2 studies on those aged ≥55 years. The sample sizes of the included studies varied from 8 to 160 participants. A total of 8 studies included male and female participants, with one study focused exclusively on women and another exclusively on men (Table 1).

**Table 1:** Study characteristics.

Author and year	Country	Age (years)	Sex	Disease focus	Sample size	Study design
Ashe et al (2015^a^) [35]	Canada	55-70	F^b^	None	25	2-arm RCT^c^
Lyons et al (2017^a^) [37]	United States	55-79	F and M^d^	None	40	2-arm RCT
Rosenberg et al (2017) [43]	United States	＞60	F and M	Obesity	10	N-of-1 trial
Mackey et al (2019) [44]	Canada	＞60	M	None	58	2-arm RCT
Recio-Rodríguez et al (2019^e^) [39]	Spain	65-80	F and M	None	160	2-arm RCT
Li et al (2020^a^) [42]	United States	65-85	F and M	None	8	Pre-post trial study
Rosenberg et al (2020) [41]	United States	60-89	F and M	Obesity	60	2-arm RCT
Blair et al (2021^a^) [36]	United States	60-84	F and M	CS^e^	54	3-arm RCT
Pinto et al (2021^a^) [38]	United States	≥65	F and M	CS	20	2-arm RCT and pre-post trial
Recio-Rodríguez et al (2022) [40]	Spain	65-80	F and M	None	157	2-arm RCT

^a^Pilot study.

^b^F: female.

^c^RCT: randomized controlled trial.

^d^M: male.

^e^Study protocol.

^f^CS: cancer survivor.

### Intervention Characteristics

Of the 10 included studies [35-44], interventions were conducted in community settings (n=3), participants’ homes (n=3), health care centers (n=2), clinics (n=1), and hospitals (n=1). These interventions ranged from a minimum duration of 25 days to a maximum of 6 months. The most common intervention frequency was once daily (n=7), while 4 studies used interventions at different time intervals, such as every 15, 20, or 30 minutes. In 2 studies, the interventions occurred weekly (n=2). One study recommended 150 minutes of moderate-to-vigorous physical activity per week. Another study conducted 1 session per week for 4 weeks, followed by monthly sessions for 5 months. In addition, one study [44] implemented the intervention 5 times per week for 30 minutes per session or 3 times per week for 20 minutes per session. The most common intervention duration was 12 weeks (n=4), followed by 3 months (n=2). Other intervention durations included 25 days, 4 weeks, 13 weeks, and 6 months. The studies were conducted in general populations (n=6), and others had a disease-specific focus (n=2). Objective sedentary behavior assessment tools, such as accelerometers, were used in 8 studies, while subjective sedentary behavior assessment tools, such as questionnaires, were used in 2. The primary outcomes measured were sedentary behavior in 3 studies. The interventions resulted in decreased sedentary behavior (n=3). Study designs varied across the included studies: 8 studies [35-41,44] used a 2-arm or 3-arm RCT design, 2 studies [38,42] used a pre-post trials design, and one study [43] used an N-of-1 trial design (Tables 1 and 2). The control conditions included no intervention (n=2), only health-related information (n=2), nutritional counseling (n=1), brief counseling, and informative leaflets (n=1; Table 2).

**Table 2:** Intervention details and assessment tools of mHealth interventions for sedentary behavior in older adults.

Author and year	Control group	Intervention site	Intervention frequency	Intervention duration	Intervention device	Assessment tool
Ashe et al (2015^a^) [35]	Health-related information only	Community	Once a week for 4 weeks, then once a month for 5 months, 10-15 minutes each	3 and 6 months	Smartphone	ActiGraph GT3X+^b^
Lyons et al (2017^a^) [37]	No intervention	Home	Daily and 1-hour intervals	12 weeks	iPad	ActivPAL^c^
Rosenberg et al (2017) [43]	No control group	Clinic	Daily and 15- or 20-minute intervals	25 days	Smartphone	ActivPAL
Mackey et al (2019) [44]	Did not receive any intervention	Community	At least 150 minutes of moderate-to-vigorous intensity physical activity per week	12 weeks	iPad	ActiGraph GT3X+
Recio-Rodríguez et al (2019^d^) [39]	Nutritional counseling, brief counseling, informative leaflet	Health care Center	5 times per week and 30 minutes per session, or 3 times per week and 20 minutes per session	3 months	Smartphone	Questionnaire
Li et al (2020^a^) [42]	No control group	Community	Daily and /90-minute intervals	4 weeks	Smartwatch	ActiGraph GT3X+
Rosenberg et al (2020) [41]	Healthy living (did not include sedentary behavior)	Hospital	Daily and 15-minute intervals	12 weeks	Smartphone	ActivPAL
Blair et al (2021^a^) [36]	No intervention	Home	Daily and 30-minute intervals	13 weeks	Smartphone	ActivPAL
Pinto et al (2021^a^) [38]	Tailored Step Goal Program and Educational Session	Home	Daily and NA^e^	12 weeks	Smartphone	ActiGraph GT3X+
Recio-Rodríguez et al (2022) [40]	Received nutritional and physical activity advice	Health care Center	Daily and NA^e^	3 months	Smartphone	Questionnaire

^a^Pilot study.

^b^Accelerometer: ActiGraph GT3X.

^c^Inclinometer: ActivPAL.

^d^Study protocol.

^e^NA: not available.

### Leveraging Information and Communication Technology for Enhanced Intervention Engagement

The included studies targeting sedentary behaviors in older adults used the latest technology, synchronizing MHAs on tablets and smartphones with wearable devices. These smart devices included smartphones (n=7), tablets (n=2), and smartwatches (n=1). For example, Ashe et al [35] used smartwatches to record step counts, which were then used to calculate the increases in steps during training sessions for older adults. Lyons et al [37] and Li et al [42] synchronized MHAs on tablets with fitness bands to monitor sedentary behavior in older adults. In terms of control groups, there were various protocols implemented across different studies as shown in Table 2.

### Theoretical Framework and Behavior Change Techniques

Among the 10 studies included in this review, 5 [35,38,41-43] extensively applied various theories to their mHealth intervention studies by focusing on sedentary behavior and physical activity among older adults. However, behavior change theoretical frameworks in mHealth interventions for sedentary behavior among older adults remain underused. These primarily include the habit formation theory [43], self-efficacy theory [42], the social cognitive theory [35], and the social-ecological theory [35] (Table 3).

**Table 3:** Theoretical framework, main assessment indicators, and outcomes in mHealth interventions for sedentary behavior in older adults.

Author and year	Theory	Main assessment indicator	Outcome
Ashe et al (2015^a^) [35]	SET^b^/SCT^c^	PA^d^	No statistically significant change in SB^e^, MVPA^f^, daily steps increases (*P*=.040)
Lyons et al (2017^a^) [37]	N/A^g^	PA	Small effects on increasing stepping time per day (Cohen *d*=0.35), steps per day (*d*=0.26), and reducing sitting time per day (*d*=0.21), body fat (*d*=0.17), and weight (*d*=0.33)
Rosenberg et al (2017) [43]	HFT^h^	SB	Breaks from sitting increase (*P=*.04)
Mackey et al (2019) [44]	N/A	PA	No statistically significant change in SB; steps increased by 1140 steps/day (95% CI 51-2229), MVPA increased by 9 minutes/day (95% CI −0.21 to 18.20)
Recio-Rodríguez et al (2019^i^) [39]	N/A	PA	N/A
Li et al (2020) [42]^i^	ST^j^	PA/SD	SB decrease (*P＜*.01), PA increase (*P*=.02), no change in sleep
Rosenberg et al (2020) [41]	SCT/SET/HFT	SB	SB decrease (*P*=.007), no statistically significant change in health condition
Blair et al (2021^a^) [36]	SCT	SB	No statistically significant change in SB and PA
Pinto et al (2022^a^) [38]	SCT	PA	MVPA increased (Cohen *d*=0.9), steps increased (*P*=.019), no statistically significant change in SB
Recio-Rodríguez et al (2022) [40]	N/A	PA	No statistically significant change in SB and PA

^a^Pilot study.

^b^SET: social ecological theory.

^c^SCT: social cognitive theory.

^d^PA: physical activity.

^e^SB: sedentary behavior.

^f^MVPA: moderate-to-vigorous physical activity.

^g^N/A: not available.

^h^HFT: habit formation theory.

^i^Study protocol.

^j^ST: self-efficacy theory.

This review synthesized studies using BCTs in MHAs to improve sedentary behavior among older adults. A total of 6 studies [35-37,41,42,44] incorporated BCTs, while 4 did not. The categorization primarily guided by the BCT taxonomy v1 developed by Michie et al [23,45] was applied to 5 studies [35-37,41,42], while one study [44] used the CALO-RE taxonomy [46]. Lyons et al [37] used the most BCTs (n=22) such as goal setting (behavior), problem-solving, action planning, review behavior goal(s), discrepancy between current behavior and goal, feedback on behavior, and social support (unspecified). Mackey et al [44] implemented 14 BCTs in their interventions, such as action planning, barrier identification and problem-solving, feedback on performance, and social support planning. Ashe et al [35] used 13 BCTs, while Blair et al [36] used 12, with both studies focusing primarily on the BCTs of goals and planning and repetition and substitution. Li et al [42] and Rosenberg et al [41] used fewer BCTs (n=6 and n=5, respectively), with both studies incorporating the following 5 BCTs: goal setting, feedback on behavior, self-monitoring of behavior, social support, and prompts/cues. Details of the BCTs used and the number of BCT interventions in 6 studies can be found in Multimedia Appendix 3.

### Risk of Bias

**Figure 2:**
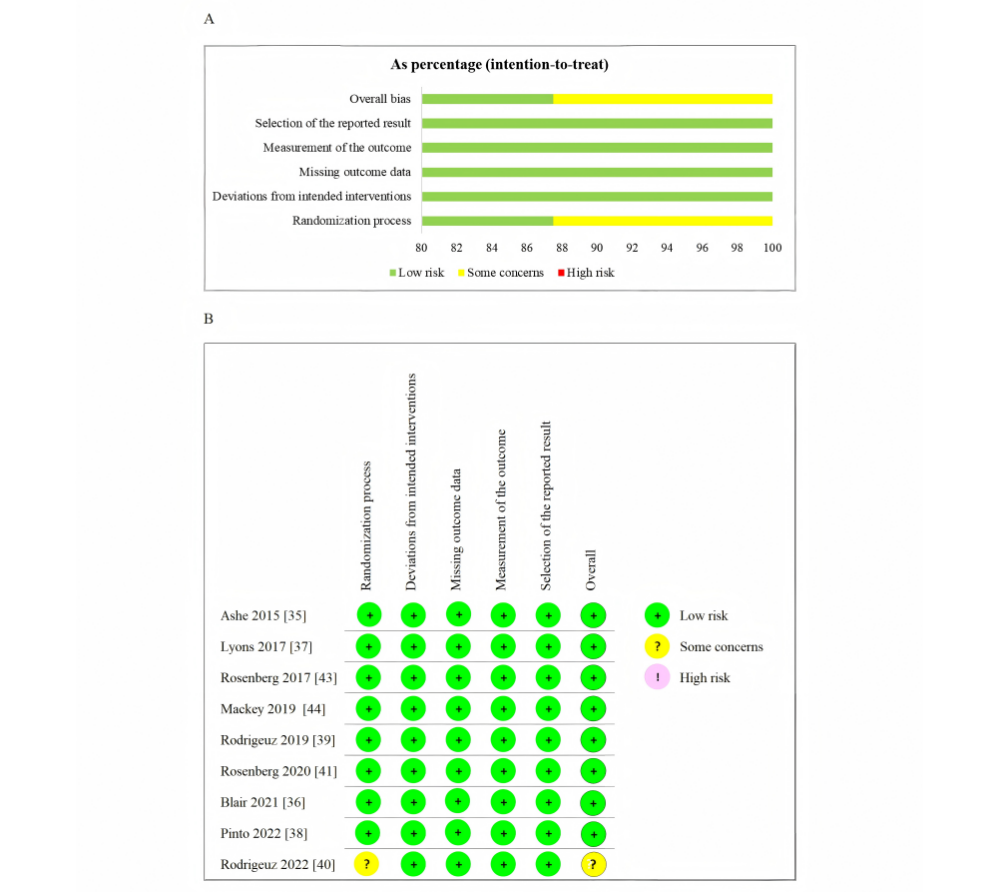
Risk of bias summary (A) and risk of bias graph (B).

The Cochrane risk-of-bias assessment results for the RCT studies are presented in Figure 2A and 2B; 8 of 9 studies [35-39,41,43,44] demonstrated a low risk of bias in all assessed domains (randomization process, deviations from intended interventions, missing outcome data, measurement of the outcome, and selection of the reported result). One study [40] showed concerns regarding the randomization process and overall risk of bias, while maintaining a low risk in the other domains. All 9 studies [35-39,41-44] indicated no significant issues in these 5 key areas, demonstrating a low risk of bias overall. The MINORS score for the nonrandomized study [42] was 11 (range 0-16), indicating a study of moderate methodological quality. Detailed MINORS score is provided in Multimedia Appendix 3.

### Intervention Effects

**Figure 3:**
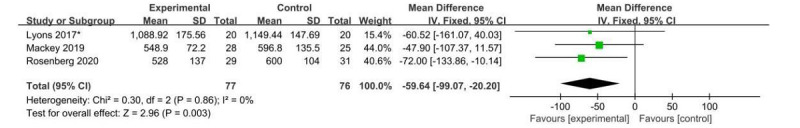
Forest plot of the effect of mHealth interventions on sitting time (min/day) [37,41,44]. The study “Lyons et al” [37] was a pilot study.

Overall, 3 studies [37,41,44], involving sitting time interventions among 153 older adults were included in the meta-analysis. A fixed-effects meta-analysis revealed a statistically significant decrease in sitting time among older adults receiving mHealth interventions compared with those receiving conventional health interventions or no intervention (WMD=59.1, 95% CI 99.1 to 20.2; Z=3.0; *P*=.003; Figure 3).

## Discussion

### Principal Findings

This systematic review and meta-analysis evaluated the effectiveness of mHealth interventions in reducing older adults’ sedentary behavior. The systematic review showed that most interventions were delivered via mobile devices in community and home settings, were primarily focused on increasing physical activity, and were implemented once daily. The meta-analysis found a significant pooled estimate of 59.1 minutes per day (95% CI 99.1 to 20.2), indicating that mHealth interventions can notably decrease sedentary behavior in older adults. However, variations in intervention settings, frequency, duration, target outcomes, and other factors might affect the consistency and generalizability of these findings. Furthermore, some studies either did not use BCTs or applied very few and lacked a systematic theoretical framework. Notably, half of the included studies were conducted in the past 5 years, reflecting a growing trend in such interventions. Nevertheless, despite their high methodological quality, the studies had relatively small sample sizes and lacked representation from developing countries, as all included studies were conducted in high-income countries.

A total of 3 studies [37,41,44] in the meta-analysis had common features: using iPads or smartphones, having interventions lasting for 15 minutes or 1 hour, promoting recommended physical activity goals, lasting for 12 weeks, applying objective assessment tools, and including BCTs related to goals and planning, feedback and monitoring, social support, and associations. Overall, 2 studies used iPads [37,44], while one used a smartphone [41], suggesting that both device types are effective. In 2 studies [37,41], daily interventions were conducted, with 1-hour or 20-minute intervals for mobile prompts to disrupt sedentary behavior. One study [44] recommended at least 150 minutes of weekly moderate-to-vigorous physical activity without specifying the distribution of these minutes. Thus, intervention frequencies of 20-minute and 1-hour intervals, or similar activity-promoting goals, may be effective. Twelve-week interventions demonstrated significant effects; only one study [35] explored the effect over 6 months. All 3 studies [37,41,44] used objective assessment tools. Two studies [37,41] applied ActivePAL, which is considered the gold standard for measuring sitting time [47], and one used ActiGraph GT3X+ [44]. Being more sensitive to sedentary behavior changes than accelerometers, ActivePAL is widely regarded as one of the most effective measurement tools [43,48-50]. Thus, the sedentary time data in these studies were relatively accurate.

Three studies [37,41,44] were conducted in diverse settings: a home [37], a community [44], and a hospital [41], in all of which sedentary behavior was effectively reduced. Only one study [41] used a theoretical framework with sedentary behavior as the primary target; the other 2 [37,44] focused on physical activity. A previous study [51] showed that interventions solely targeting sedentary behavior were more effective than combined interventions. Notably, many studies [35-40,42,44] concluded that sedentary behavior and physical activity interventions often reported sedentary behavior as a secondary outcome. An analysis of 3 studies on mHealth intervention found that replacing sedentary behavior with physical activity as a means of reducing sedentary behavior did not significantly reduce sitting time in older adults [36]; this was in line with another study [41]. Nevertheless, 2 RCTs [41,43] using mHealth to interrupt sitting time in older adults achieved positive results, highlighting the importance of targeted sedentary behavior reduction interventions.

Furthermore, all 3 studies [37,41,44] integrated multiple BCTs related to goals, planning, feedback, monitoring, social support, and associations. Multicomponent BCT interventions generally yield better results than single-technique interventions in sedentary behavior reduction [51]. Core techniques such as goal setting, self-monitoring, and feedback were part of an effective intervention associated with sedentary time reduction, as supported by existing studies [37,52]. mHealth tools, with real-time monitoring, can prompt older adults to take action during prolonged sedentary periods [37,52]. They also feature persuasive elements, such as personalized motivational messages and progress tracking, to maintain engagement [53]. With highly customizable features, mHealth can be tailored to older adults’ needs and preferences, enhancing the intervention’s effectiveness [54]. This comprehensive approach makes mHealth a potentially more effective tool for reducing sedentary behavior compared with other digital interventions [18].

Previous meta-analyses [55-57] emphasized the advantages of theory-based interventions, yet few specifically addressed sitting time. In studies targeting sedentary behavior, Rosenberg et al [41,43] highlighted the significance of leveraging theories such as the social ecological theory, social cognitive theory, and habit formation theory to facilitate behavior change. Sedentary behavior is shaped by multiple factors, including physiological, psychological, social, environmental, and policy aspects [58]. The behavior change wheel (BCW) [45] should be applied in interventions, particularly those targeting both sedentary behavior and physical activity. The BCW offers a structured framework for designing behavior change interventions. Its 3-layer wheel model focuses on capability, opportunity, and motivation (inner layer), 9 intervention functions (middle layer), and 7 policy categories (outer layer) [52]. The BCW considers individual characteristics (behavior sources), intervention methods (functions), and social factors (policy categories) as crucial for intervention success [52]. Michie et al [52] proposed 93 BCTs, grouped into 16 categories based on BCW, to facilitate an insightful understanding of behavior change mechanisms and more precise tailoring of interventions. A systematic review showcased that theory-based, multi-BCTs are most effective for health behavior change [59]. A previous study, including one using BCW [60], analyzed sedentary behavior mechanisms and designed interventions for occupational populations and confirmed the effectiveness of BCTs in reducing sedentary behavior and the practical value of BCW.

### Comparison With Other Studies

A meta-analysis of relatively healthy community-dwelling older adults [18] showed that mHealth interventions might reduce sedentary time and promote physical activity in the short term (≤3 months). However, nonsignificant results and the inclusion of high-risk studies based on ROB 1.0. Similarly, another meta-analysis with 21 trials [61] had limitations, with only one study on older adults and combined eHealth interventions, potentially diluting the impact of mHealth interventions on sedentary behavior. Our study addresses these limitations in several ways. First, we expanded intervention settings beyond the community to include home, clinical, and health care center settings. Second, we included older adults with a broader range of health conditions such as cancer survivor and obesity, not just healthy ones. Third, we used the revised Cochrane ROB 2.0 tool, with all included studies demonstrating low bias risk, which is more rigorous than using ROB 1.0 before. Finally, our study focused solely on older adults. Therefore, by isolating mHealth interventions, our study provided more substantial evidence of the effectiveness of mHealth in reducing older adults’ sedentary behavior.

Overall, future intervention guidelines should be multifaceted to effectively address sedentary behavior in older adults. First, leveraging the BCW could help in comprehensively understanding and tackling the determinants of sedentary behavior. Second, mHealth intervention goals should be tailored to older adults, focusing on altering sedentary behavior and incorporating BCTs related to goals and planning, feedback and monitoring, social support, and associations. Thirdly, exploring different BCT combinations’ effects on reducing sedentary behavior is essential for promoting and maintaining positive behavior change. Fourthly, interventions could use devices such as iPads or smartphones, incorporate daily prompts at intervals of 20 minutes to 1 hour, and last at least 12 weeks, with extended follow-up durations to ensure effectiveness and sustainability. Furthermore, future studies, particularly in developing countries, should focus on specific target populations, explore different intervention durations, and assess long-term effects. Finally, comprehensive measurement of specific sedentary behavior in older adults should be emphasized, alongside the standardization of measurement units (eg, minutes per day, percentage of the day, and hours per week) to improve the universality and accuracy of research findings.

### Limitations

This study has certain limitations. Firstly, it only includes articles published in English, which limits the global comprehensiveness of our research outcomes. Secondly, the lack of uniformity in measurement units used by these tools presents challenges in conversion. Thirdly, most existing studies focus on combining physical activity with sedentary behavior, predominantly emphasizing the augmentation of physical activity. Literature directly addressing the reduction of sedentary behavior in older adults is relatively rare, making it challenging to ascertain the distinct role of reducing sedentary behavior. Finally, since this field is rapidly evolving, new studies may have been published after our search cutoff date, potentially influencing the current evidence base.

### Conclusions

This systematic review and meta-analysis evaluated mHealth interventions for managing sedentary behavior in older adults. The findings indicate that mHealth interventions significantly reduce sedentary behavior in this demographic. Given the potential of mHealth intervention strategies that incorporate specific frequencies, durations, dedicated mobile monitoring devices, and tailored BCTs, these findings underscore the critical role of mHealth in translating research into effective public health strategies for older adults.

## Appendix

Multimedia Appendix 1 PRISMA checklist.

Multimedia Appendix 2 Search strategy.

Multimedia Appendix 3 Summary of BCTs, number of intervention BCTs, and MINORS score.

## Abbreviations

BCT: behavioral change techniques
BCW: behavior change wheel
MeSH: Medical Subject Headings
MHA: mHealth apps
mHealth: mobile health
MINORS: Methodological Index for Non-Randomized Studies
PRISMA: Preferred Reporting Items for Systematic Reviews and Meta-Analysis
PROSPERO: Prospective Register of Systematic Reviews
RCT: randomized controlled trial
ROB 2: Cochrane Risk of Bias 2 (ROB 2) Tool
WHO: World Health Organization
WMD: weighted mean difference
