# Supplementary material for: Effectiveness of Mobile Health Interventions for Reducing Sitting Time in Older Adults: Systematic Review and Meta-Analysis
Source: J Med Internet Res. 2025 May 8;27:e60889. doi: 10.2196/60889 (PMC12101137; doi:10.2196/60889)
Supplement: Multimedia Appendix 2 [file jmir_v27i1e60889_app2.docx]

**Effectiveness of mHealth Sitting Time Interventions in Older Adults: A Systematic Review and Meta-Analysis**

Table 1. Study Characteristics

| Author/Year | Country | Age (years) | Sex | Disease Focus | Sample Size | Study Design |
| --- | --- | --- | --- | --- | --- | --- |
| Ashe 2015 ^a^ | Canada | 55~70 | F | None | 25 | Two-arm RCT |
| Lyons 2017 ^a^ | USA | 55~79 | F/M | None | 40 | Two-arm RCT |
| Rosenberg 2017 | USA | ＞60 | F/M | Obesity | 10 | N-of-1 trial |
| Mackey 2019 | Canada | ＞60 | M | None | 58 | Two-arm RCT |
| Rodríguez 2019 ^b^ | Spain | 65~80 | F/M | None | 160 | Two-arm RCT |
| Li 2020 ^a^ | USA | 65~85 | F/M | None | 8 | Pre-posttrial study |
| Rosenberg 2020 | USA | 60~89 | F/M | Obesity | 60 | Two-arm RCT |
| Blair 2021 ^a^ | USA | 60~84 | F/M | CS | 54 | 3-arm RCT |
| Pinto 2022 ^a^ | USA | ≥65 | F/M | CS | 20 | Two-arm RCT and pre-post trial |
| Rodríguez 2022 | Spain | 65-80 | F/M | None | 157 | Two-arm RCT |

Note: ^a^ = Pilot study; ^b^ = Study protocol; F = Female; M = Male; CS = Cancer Survivor; USA = United States of America.

Table 2. Intervention Details and Assessment Tools of mHealth Interventions for Sedentary Behavior in Older Adults

| Author/Year | Control group | Intervention site | Intervention frequency | Intervention duration | Intervention device | Assessment tool |
| --- | --- | --- | --- | --- | --- | --- |
| Ashe 2015 ^a^ | Health-related information only | Community | Once a week for 4 weeks, then once a month for 5 months, 10-15 minutes each | 3 and 6 months | Smartphone | ActiGraph GT3X+ |
| Lyons 2017 ^a^ | No intervention | Home | Daily/1-hour intervals | 12 weeks | iPad | ActivPAL |
| Rosenberg 2017 | No control group | Clinic | Daily/15 or 20 minutes intervals | 25 days | Smartphone | ActivPAL |
| Mackey 2019 | Did not receive any intervention | Community | At least 150 minutes of moderate to vigorous intensity physical activity per week | 12 weeks | iPad | ActiGraph GT3X+ |
| Rodríguez 2019 ^b^ | Nutritional counseling, brief counseling, informative leaflet | Healthcare Center | 5 times per week / 30 minutes per session, or 3 times per week / 20 minutes per session | 3 months | Smartphone | Questionnaire |
| Li 2020 ^a^ | No control group | Community | Daily/90 minutes intervals | 4 weeks | Smartwatch | ActiGraph GT3X+ |
| Rosenberg 2020 | Healthy living (did not include sedentary behavior) | Hospital | Daily/15-minute intervals | 12 weeks | Smartphone | ActivPAL |
| Blair 2021 ^a^ | No intervention | Home | Daily/30 minutes intervals | 13 weeks | Smartphone | ActivPAL |
| Pinto 2022 ^a^ | Tailored Step Goal Program and Educational Session | Home | Daily/NA | 12 weeks | Smartphone | ActiGraph GT3X+ |
| Rodríguez 2022 | Received nutritional and physical activity advice | Healthcare Center | Daily/NA | 3 months | Smartphone | Questionnaire |

Note: ^a^ = Pilot study; ^b^= Study protocol; NA = Not Available; Acceleromete r= ActiGraph GT3X; Inclinometer = ActivPAL.

Table 3. Theoretical Framework, Main Assessment Indicators, and Outcomes in mHealth Interventions for Sedentary Behavior in Older Adults

| Author | Theory | Main assessment indicator | Outcome |
| --- | --- | --- | --- |
| Ashe 2015 ^a^ | SET/SCT | PA | No statistically significant change in SB, MVPA, daily steps increases (*p*=0.040) |
| Lyons 2017 ^a^ | NA | PA | Small effects on increasing stepping time per day (Cohen's d=0.35), steps per day (d=0.26), and reducing sitting time per day (d=0.21), body fat (d=0.17), and weight (d=0.33) |
| Rosenberg 2017 | HFT | SB | Breaks from sitting increase (*p=*0.04) |
| Mackey 2019 | NA | PA | No statistically significant change in SB; steps increased by 1140 steps/day (95% CI: 51-2229), MVPA increased by 9.0 minutes/day (95% CI: -0.21-18.20) |
| Rodríguez 2019 ^b^ | NA | PA | NA |
| Li 2020* | ST | PA/SD | SB decrease (*p＜*0.01), PA increase (*p*=0.02), no change in sleep |
| Rosenberg 2020 | SCT/SET/HFT | SB | SB decrease (*p*=0.007), no statistically significant change in health condition |
| Blair 2021 ^a^ | SCT | SB | No statistically significant change in SB and PA |
| Pinto 2022 ^a^ | SCT | PA | MVPA increased (Cohen’s d = 0.9), steps increased (*p*=0.019), no statistically significant change in SB |
| Rodríguez 2022 | NA | PA | No statistically significant change in SB and PA |

Note: ^a^= Pilot study; ^b^= Study protocol; SB = Sedentary Behavior; PA = Physical Activity; MVPA = Moderate-to-Vigorous Physical Activity; SD = Sleep Duration; SET = Social Ecological Theory; SCT = Social Cognitive Theory; HFT = Habit Formation Theory; ST = Self-Efficacy Theory; ET = Ecological Theory; NA = Not Available.
